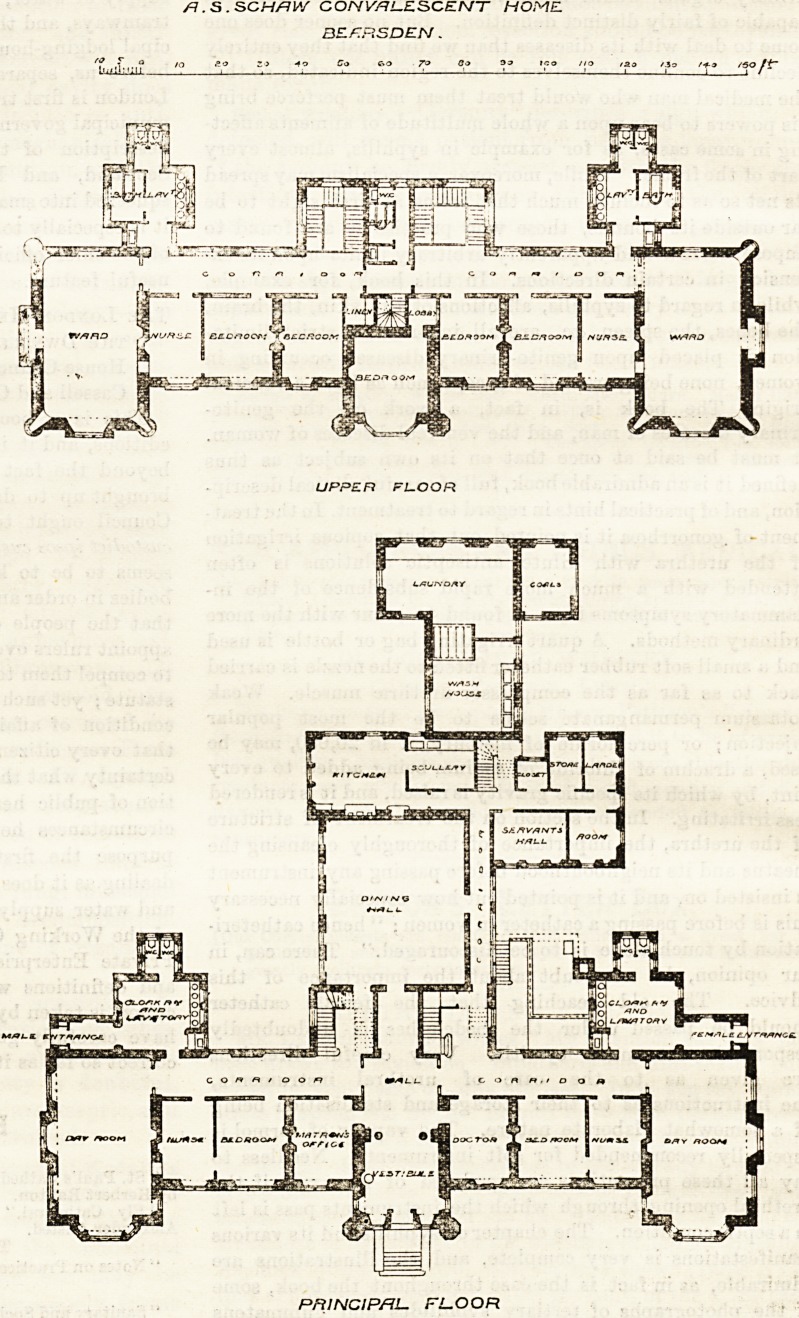# Hospital Construction

**Published:** 1897-06-19

**Authors:** 


### The Institutional Workshop.

### HOSPITAL CONSTRUCTION.

### SCHAW CONVALESCENT HOME, BEARSDEN.

This institution, erected in memory of her brother by Miss Schaw, of Glasgow, stands in grounds of more than 12 acres in an elevated and commanding position. Accommodation is provided for 50 patients, 25 of each sex, and the accompanying plans show the general arrangement of the top and principal floors.

From the main entrance under the central tower of the principal block, side-lighted corridors, 10 feet wide, run right and left to the women's and men's rooms respectively. These corridors are repeated on the upper floors; separate staircases for the two sexes connecting them throughout the building.

The block containing the dining-hall, kitchen, and its appurtenances runs back at right angles to the axis of the main building. Separate entrances from the men's and women's corridors are provided to the dining-hall, which the kitchen and servants' rooms immediately adjoin. The laundry forms a one-storied building at the rear of the kitchen wing.

The ground floor of the principal building has the matron's and doctor's rooms left and right of the central entrance hall. Next to these a bedroom, 20 feet by 17 feet, for each sex opens out of the corridors, commanded by the nurses' rooms, which adjoin the day rooms. These stand across the end of the corridors, and measure 39 feet by 20 feet 6 inches each. Good cloak-room and lavatory accommodation opens off the corridors at each end (the w.c.'s being carefully isolated), and well-arranged entrances for each sex from the grounds are provided in connection with the cloak-rooms.

In the basement a smoking-room for the men and a work-room for the women are stated to be provided. The upper floors are devoted to dormitory accommodation. Large wards are placed over each of the day-rooms, and two smaller wards and a nurses' room open out of those portions of the corridor appropriated to each sex on each floor, the corridors being divided by a screen.

Bath-rooms, w.c.'s, and lavatory accommodation, carefully and properly isolated, are provided on each floor in separate blocks over the cloak-rooms, &c., on the principal floor. In the centre between the staircases on the top floor a w.c., bath-room, and sink are shown opening off a separate passage. Probably these are designed for the use of the staff; but the bath-room and sink do not appear to be provided with any direct light and air. This appears to be a blot in a building exceptionally well arranged in other respects, while on the top floor the architect would seem to have sacrificed to the artistic treatment of the exterior the practical arrangement of windows for ward purposes. The window to the scullery on the principal floor appears cramped, but possibly there is top-light available here. For general simplicity of arrangement, airiness, and spaciousness, the Schaw Convalescent Home appears worthy of much praise and careful study.

## Figures and Tables

**Figure f1:**